# Endoscopic repair of CSF rhinorrhea: experience of 44 cases

**DOI:** 10.1016/S1808-8694(15)31202-7

**Published:** 2015-10-20

**Authors:** Bernardo Cunha Araujo Filho, Ossamu Butugan, Francini Grecco de Melo Pádua, Richard Louis Voegels

**Affiliations:** 1 Otorhinolaryngologist (residence program at HCFMUSP), Specialist in Otorhinolaryngology, SBORL, Ph.D. studies under course, Division of Clinical Otorhinolaryngology, HCFMUSP.; 2 Associate Professor, Discipline of Otorhinolaryngology, Medical School, University of Sao Paulo.; 3 Fellow in endoscopic surgery, Discipline of Otorhinolaryngology, FMUSP; Fellow in endoscopic surgery, Discipline of Otorhinolaryngology, University of Graz, Austria; Post-graduate studies in Otorhinolaryngology under course, Discipline of Otorhinolaryngology, FMUSP.; 4 Associate Professor, Discipline of Otorhinolaryngology, Medical School, University of Sao Paulo.

**Keywords:** cerebrospinal fluid leakage, endoscopic surgery, endoscope

## Abstract

Cerebrospinal fluid (CSF) rhinorrhea is a leakage of fluid from the subarachnoid space to the frontal, sphenoidal or ethmoidal sinuses. CSF rhinorrhea is a known potential complication with significant morbidity and mortality. It may present a significant challenge in diagnosis, localization and management. **Study design:** series study. **Material and method:** Between 1993 and 2004, 44 patients with cerebrospinal rhinorrhea were operated on using intranasal endoscopic approach in the University Hospital of the University of Sao Paulo, Medical School. The charts of all patients treated in our hospital were reviewed. **Results:** Forty-four patients, 16 women (36%) and twenty-eight men (64%), were included in the study. Patients’ ages ranged from 2 to 68 years (mean: 40.3 years). Etiology, site of leakage, diagnosis, technique, cause of failure and follow-up are discussed. **Conclusion:** The authors concluded that transnasal endoscopic surgery for CSF rhinorrhea had high success rate, low morbidity and stable long-term results.

## INTRODUCTION

Cerebrospinal fluid (CSF) rhinorrhea was firstly described by Galen, 200 B.C. 1, 2. Saintclair Thompson reported the first series of patients with spontaneous leakage in 1889^1^. Many attempts to correct a SF leak were done in the 20^th^ Century, although the first well-succeeded surgical approach was attributed to Dandy in 1926, when he sutured the fascia lata over dural defect, on back of the posterior wall of the frontal sinus, by intracranial route^2^. In 1964, Vrabec and Hallberg described endonasal approach to repair a SF leak in the cribriform lamina^2^, ^3^.

Fluid leakage may be expressed by several symptoms and/or signs, although rhinorrhea^4^ is the most frequent one. In addition, presence of SF may be a risky condition for patient and onset of infections in the central nervous system, such as meningitis^2^, ^4^. Thus, in the absence of spontaneous resolution, a corrective procedure should be performed. In 1981, when Wigand repaired fistulas using an endoscope, this approach became widely adopted^4^.

Today, with improvements of skull base surgeries and the introduction of functional endoscopic surgery of paranasal sinuses in the otorhinolaryngology’s routine, there has been increased incidence of severe complications, such as the cerebrospinal fluid leakage^5^. Thus, endonasal duraplasty has been successfully used to repair several dura mater lesions with low patient’s morbidity rate 2, 6, 7.

In this article, we present the experience with repair of 44 CSF cases using endoscope.

## MATERIALS AND METHODS

Between 1993 and 2004, 44 patients diagnosed with cerebrospinal fluid rhinorrhea were submitted to endonasal endoscopic surgery in *Hospital das Clínicas,* Medical School, *Universidade de São Paulo.* All patients’ medical files were revised in our service. Forty-four patients – 16 (36%) women and 28 (64%) men were evaluated in the study. Age varied from 2 to 69 years (mean: 40.3). Information related to cause, site of dural lesion, repairing technique, complications and follow-up were collected from the files (Table 1).

**Table 1:** Cause of SF leakage site in patients treated by endonasal technique (n=44)

**Site of ethmoid fistula**	
Cause of CSF + Cribriform	Frontal Sphenoid
Post-operatory	66
Trauma	862
Congenital Fistula	1367
(Meningoencephalocele)	
Spontaneous	945
Post-exeresis of hypophysis adenoma	88
TOTAL	4422202

**Figure 1:**
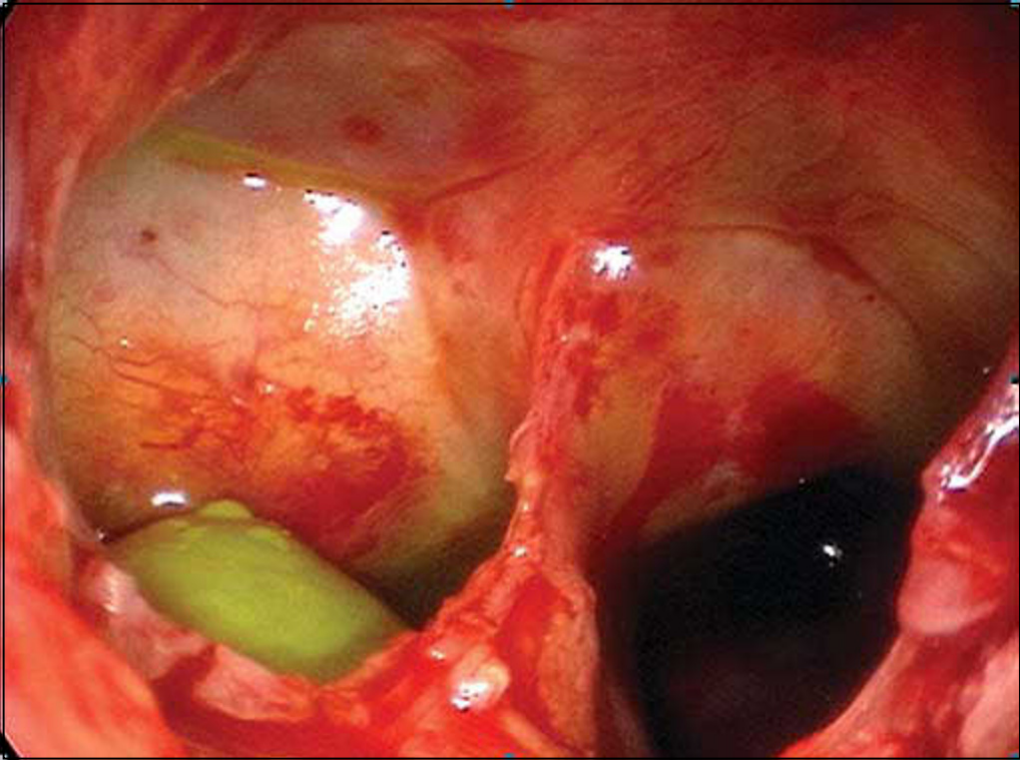
Fistula of right sphenoid sinus, drainage of CSF with fluorescein marker (green).

**Figure 2:**
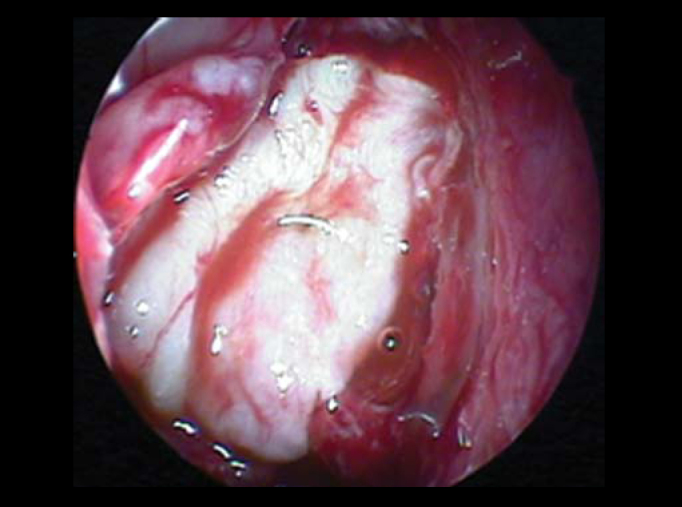
Flap mucous-free perichondrium using “onlay” technique.

In all cases, fluorescein was applied by lumbar puncture, right before surgery to facilitate identification of dural lesion and to monitor the success rate during the procedure (Figure 1). 5%-sterile fluorescein was injected in intrathecal region by lumbar puncture. We have used a 0.1ml solution/10 kg of body mass (not exceeding 1 ml) diluted in distilled water up to 10 ml. “Onlay” and “Underlay” techniques were used for correction of dural defect. All fistulas were repaired with connective tissues, such as free-mucosa flaps (Figure 2), pediculated flaps, liofilized dura, bovine pericardium or fascia lata and were fixed with fibrin glue and Surgicel®. In order to check success or failure rate during procedure, clinical exams, endoscopic controls and, when necessary, nasal endoscopic approach using fluorescein, were performed at late postoperative phase.

## RESULTS

**Figure 3:**
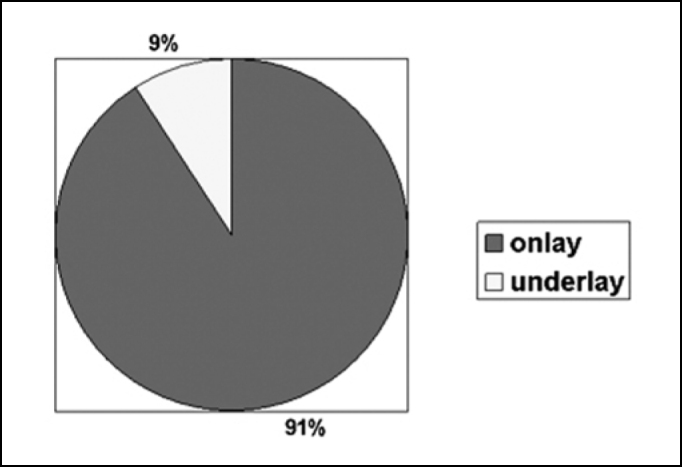
Technique used for fistula repair.

Causes of cerebrospinal fluid fistula and the sites in our series are listed in Table 1. The most frequent site of fistulas was the ethmoidal cribriform area. Meningoencephalocele was the most prevalent cause of fistula. “Underlay” technique was used in 4 cases (9%), while “onlay” was performed in 40 cases (91%) (Figure 3). “Onlay” technique was used in the patients who were also undergoing “underlay” technique. Surgicel® was used to fill the sphenoid sinus when SF was detected.

Liofilized dura was used in 27 cases, while mucous flap was used in 39 cases. Fascia lata was used in 2 cases. External lumbar shunt (ELS) was performed in 35 patients.

Patients remained in relative rest and in decubitus with maximal inclination of 10° for 5 days, on average, and ELS was maintained for 4 days. Laxative diet and instructions to avoid Valsalva were recommended to patients. Nasal packing was not used and prophylactic antibiotic was introduced during anesthesia induction. We preferred using ceftriaxone, third-generation cephalosporin with penetration in the hematoencephalic barrier.

Postoperative follow-up varied from 2 to 102 months (mean: 39.4 months).

The first attempts of surgical SF correction had 9 failures. Among these patients, seven were submitted to another endoscopic approach, while 2 cases presenting extensive bone failure were submitted to craniotomy for fistula repair by the neurosurgery service.

Only one patient developed postoperative meningitis, although with complete recovery after antibiotic therapy. One patient developed hyper-drainage of cerebrospinal fluid caused by an unleveled placement of the collector bag. All other patients improved without further complications.

## DISCUSSION

**Figure 4:**
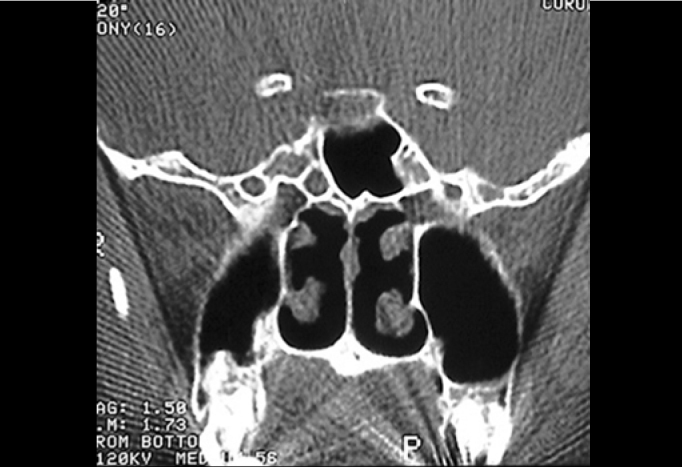
Computed Tomography of patient with CSF fistula in meningocele of sphenoid sinus.

Communication between the subarachnoid space and the nasal cavity is called cerebrospinal fluid rhinorrhea^1^, ^8^, which can occur directly from the anterior cranial fossa and the nasal cavity, or indirectly from the middle and posterior fossa through the auditory tube^1^. CSF’s may be classified into traumatic and non-traumatic^1^, ^9^. In our series, 14 (32%) of SL’s were caused by trauma (6 surgical traumas and 8 non-surgical traumas), and the most affected site was the cribriform lamina and the posterior ethmoidal region, where bone is thinner and dura’s adherence to this bone is stronger^1^ (Figure 4). Presence of spontaneous SF is variable in the literature, ranging from 4 to 39%. Our findings were similar to those reported by Wax et al. (1997), in which spontaneous leakages represented 29.5% in our population. Origin of spontaneous leakage remains unclear, however some studies presented explanations such as: a congenital disorder; representation of a small meningocele eroding through bone or deriving from a focal atrophy of olfactory nerve filaments in the cribriform lamina^1^, ^6^. During preoperative evaluation and in order to locate the lesions site, all patients were submitted to compute tomography, with axial and coronal sections of paranasal sinuses. We have not used B2-transferrin, which is the most sensitive test to confirm SF leakage^5^, since it was not available, while magnetic resonance (MRI) was requested only for suspected cases of meningoencephaloceles, differently from Nachtigal and Schick studies, which proposed MRI for all cases^2^, ^4^.

A tomocisternography was not used for inconclusive cases either; instead, intrathecal fluorescein infusion and diagnostic endoscopic visualization were preferred. All patients had history of rhinorrhea with “rock water” liquid in one nostril, while diagnosis of SF was confirmed by endoscopic visualization of this sample drained from the nasal roof. Attention is to be given to differential diagnosis with rhinitis, as suggested by Ramsden (2000), who reported the case of a 47-year woman, with bilateral rhinorrhea and no history of trauma, who was treated for many years for rhinitis when in fact it was a case of spontaneous bilateral CSF^6^.

SF correction by endonasal technique has been broadly performed and the world literature shows that it is a secure and efficient method. Localization and visualization by endoscope allow the surgeon to more easily remove the fibrous tissue and scarify the lesion’s borders, which yield greater graft adherence in the region with excellent outcomes^1^, ^10^.

There are several distinct endonasal techniques for SF correction and all have been successful^2^, ^5^. In addition, a variety of autologous and heterologous grafts derive from connective tissues. In our sample, as well as in the literature mentioned, we have not observed surgical failures associated with a specific type of graft used. According to Schick (2001) and Hao (1996), incomplete exposure and large bone defect were prevalent factors in therapeutic failures.

**Figure 5:**
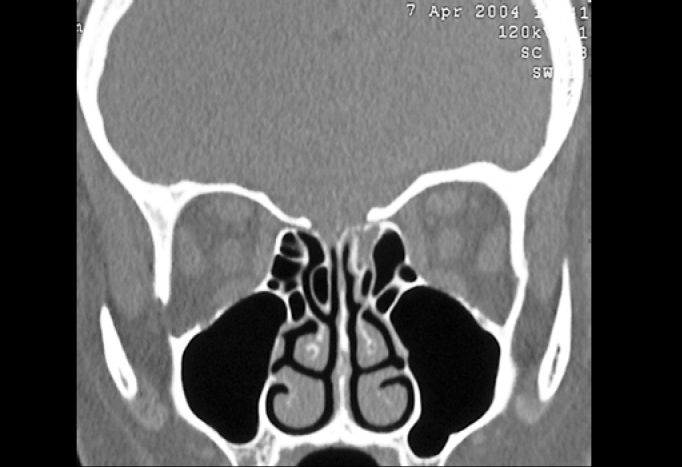
Computed Tomography of patient with CSF fistula in cribriform lamina during left ethmoidal sinusectomy.

In our series, success rate of first intervention was of approximately 80%, which is similar to results reported by Schick et al. (2001), Burns et al. (1996), Hao et al. (1996) and Ryet et al. Failures were probably due to large defects, such as hernia of meningoencephalic sac at the cribriform area or in the sphenoid sinus (Figure 5), or to extensive front-basal fractures. Non-recognition of dural defect adjacent to primary defect was the cause of failure in the series of Hao et al. (1997). In these cases, due to high deficit of fistulas, there was impairment in graft placement and insufficient adherence.

We observed that postoperative fistulas from endoscopic surgery affected the cribriform lamina and posterior ethmoid, a fragile region in the skull base^4^, ^10^ to which attention should be given in suspected cases of CSF.

Meningitis is the most feared life-risk complication in patients with CSF^11^. Studies demonstrate incidence in up to 40% of patients. We only had one case of meningitis associated with large bone defect, which corroborates low morbidity of this endonasal technique. Postoperative patients should be carefully assisted, considering that non-observation of correct placement of the CSF collector bag was responsible for one case of hyper-drainage syndrome associated with significant headache.

Use of prophylactic antibiotics is controverisial^11^. We used antibiotics in all patients; however, Nachtigal (1999), who had not adopted a prophylactic approach, had no cases of meningitis in his 12-patient series. Among 115 patients with accidental CS leakage, Choi (1996) observed a higher incidence of meningitis in patients who underwent antibiotic prophylaxis.

## CLOSING REMARKS

We came to the conclusion that endonasal fistula repair is the approach of choice to reach high success rate and low morbidity in the treatment of cerebrospinal fluid rhinorrhea. SF leakage associated with large defects, meningoceles and secondary lesions should receive special attention, while many times technique modifications are necessary to assure continuous fistula closure.
